# Surgical implantation of a biventricular pacing system via lower half mini sternotomy

**DOI:** 10.1186/1749-8090-8-5

**Published:** 2013-01-12

**Authors:** Morteza Tavakkoli Hosseini, Aron Frederik Popov, Antonios Kourliouros, Mazin Sarsam

**Affiliations:** 1 Department of Cardiothoracic Surgery, St. George’s Hospital, BlackShaw Road, London SW17 0QT, UK; 2 Department of Thoracic and Cardiovascular Surgery, University of Goettingen, Goettingen, Germany

**Keywords:** Arrhythmia Therapy, Pace Maker, Minimally invasive incision

## Abstract

We present a case of surgical implantation of biventricular epicardial pacing leads and a defibrillating patch via lower half mini sternotomy. Although median sternotomy is routinely used for this purpose, lower half mini sternotomy could provide the surgeon with the same surgical field exposure and a faster post operative recovery.

## Background

Transvenous implantation of permanent atrial or ventricular pacing leads is the method of choice in adults. It is a well tolerated procedure that is generally performed with local anesthesia under fluoroscopic control [1]. Right heart leads are placed through the subclavian vein and superior vena cava, while left ventricular lead implant is accomplished percutaneously through coronary sinus cannulation, advancing the lead into a major cardiac vein [2]. Unfortunately, this technique is associated with long fluoroscopy times and is not applicable to all patients because of coronary sinus and coronary venous anatomy limitations [3].

The optimal pacing system implantation technique in the presence of infected endocardial pacing system or limited venous access to the heart has not yet been defined [4,5]. Surgical implantation of epicardial pacing leads is an option in children and adults who are not suitable for traditional transvenous approach. While median sternotomy is the common approach for implantation of an epicardial pacing system, other surgical approaches like left lateral thoracotomy [6], subcostal approach [7], limited lower sternotomy [8] and video-assisted thoracic surgery [1] have also been described. Although median sternotomy provides access to the surface of all chambers of the heart, it may be unnecessary for a full sternotomy. With a left thoracotomy, access to the right side of the heart is restricted and with a subcostal approach difficulty arises if one attempts to implant a defibrillating patch. The limited lower sternotomy technique described in the literature [8] only allows access to the right atrium and ventricle and not to the left side of the heart. We present a case of surgical implantation of biventricular epicardial pacing leads and defibrillating patch via lower half mini sternotomy.

## Case presentation

A 60 year old male presented with Staphylococcus aureous infection of his endocardial pacing system. His past medical history was remarkable for mild dilated cardiomyopathy, ventricular tachycardia, a biventricular endocardial pacing for cardiac resynchronization therapy, and several previous percutaneous endocardial pacing system implantations via the right and the left subclavian veins. The pacing leads and box were removed and the infection was treated with antibiotics. Due to subclavian vein stenosis and pacing box pocket infection, transvenous approach for implantation of a new pacing system proved not to be feasible. Therefore he was referred for surgical implantation of a new epicardial pacing system.

**Figure 1:**
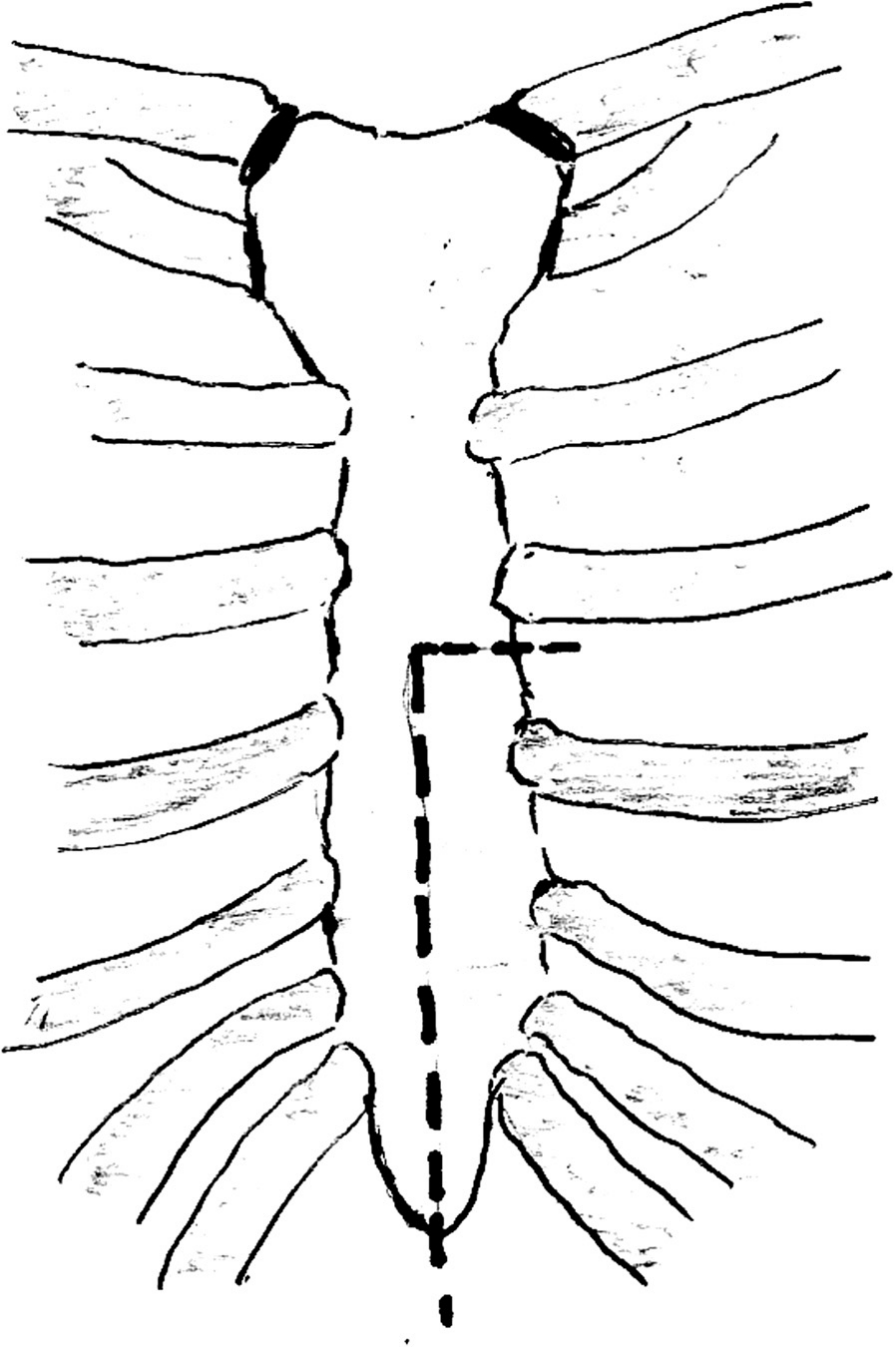
Lower half mini sternotomy.

**Figure 2:**
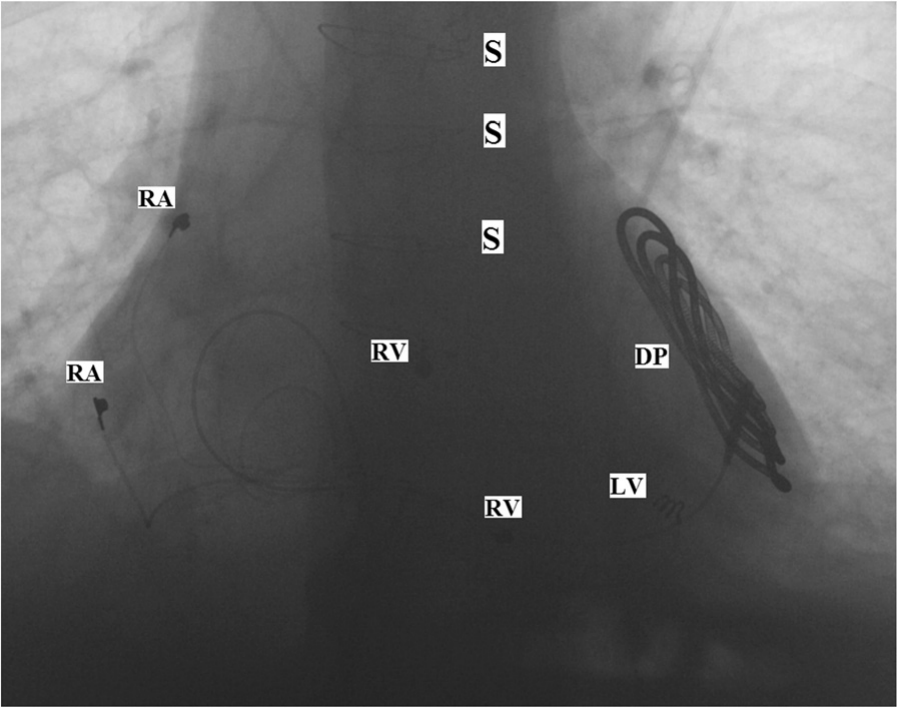
**Pacing lead positions. RA: **Right Atrial Lead, **RV: **Right Ventricular Lead, **LV: **Left Ventricular Lead, **DP: **Defibrillating Patch, **S:** Sternal Wire.

A lower half limited median sternotomy was performed starting from the xiphoid process, up to the level of the third intercostal space. A second limited transverse sternotomy was performed starting from the left third intercostal space and joining the midline sternotomy (Figure 1). The pericardium was opened in the midline, providing excellent exposure and easy access to both the right atrial appendage and the right ventricle. The right atrial leads were sutured to the surface of the right atrial appendage, while the right ventricular bipolar leads were sutured to the anterior muscular part of the right ventricle. Access to the left ventricle was achieved by gently lifting up the heart, using an epicardial tissue stabilizing system and exposing the lateral wall of the left ventricle with no hemodynamic compromise. The left ventricular lead was screwed into the proximal part of the lateral wall between the obtuse marginal arteries and then the defibrillator patch was sutured to the distal lateral wall. It was made sure that there was a good orientation between the patch and the pacing box which was subsequently inserted in the sheath of the right rectus muscle. The thresholds on the pacing leads were checked and the defibrillating patch was tested. A right subcostal incision was made and a pocket was created in the lateral side of the right rectus muscle. The pacing wires were tunneled between the right hemi-diaphragm and the lateral edge of the right rectus muscle, into the pacing box pouch and connected to the pacing box. Defibrillation threshold testing was performed, and the lowest effective threshold was found to be < 10 Jules. The sternum was approximated using interrupted stainless steel wires. Sternotomy and subcostal incisions were closed in layers. The patient had a good post operative recovery and was discharged home on day 4 after the operation. Follow-up studies showed well functioning biventricular pacing system, stable pacing lead positions (Figure 2), good biventricular synchronized function, and no pericardial effusion.

## Conclusions

The optimal pacing system implantation technique in the presence of limited venous access to the heart has not yet been defined [4,5]. Different surgical implantation techniques have been described for different clinical scenarios. Although median sternotomy provides a good access to the surface of all chambers of the heart, it may be unnecessary for a full sternotomy. The limited lower sternotomy technique described in the literature [8] only allows access to the right atrium and ventricle and not to the left side of the heart. For proper access to the left side of the heart, the mini sternotomy needs to go up to the level of the third intercostal space and then directed toward the left. This allows the surgeon to lift up the heart and have good access to the lateral wall. A smaller skin incision, a more stable sternum, and a better cosmetic result are also important. Bilateral mini thoracotomy could also be considered as an option in similar cases. Our technique could be considered for children, patients with limited venous access to the heart, patients with single ventricle disease, and patients with intracardiac shunts who cannot have a typical transvenous BiV ICD system.

Although a screw-in lead was used for the left ventricle at the time of our operation, it is now recommended to use steroid eluting bipolar leads. To provide an ideal vector, the defibrillating patch should be placed at the posterior aspect of the left ventricle and the pacing box should be implanted at the left side of the abdomen. Due to the limitations of the mini-sternotomy incision, defibrillating patch was implanted on the lateral wall and the pacing box was inserted in the right side of the abdomen. Also based on the current technology, it is now recommended to use an epicardial coil instead of a defibrillating patch.

## Consent

Written informed consent was obtained from the patient for publication of this Case report and any accompanying images. A copy of the written consent is available for review by the Editor-in-Chief of this journal.

## Competing interests

The authors declare that they have no competing interests.

## Authors’ contributions

All authors participated in the operation. MTH contributed to literature search, writing the manuscript, and its final revision. AFP gave important comments. AK contributed to searching the literature and writing the manuscript. MS contributed to case presentation, final revision, and supervision. All authors read and approved the final manuscript.
